# Exploring the Potential of a Smart Ring to Predict Postoperative Pain Outcomes in Orthopedic Surgery Patients

**DOI:** 10.3390/s24155024

**Published:** 2024-08-03

**Authors:** Michael Morimoto, Ashraf Nawari, Rada Savic, Meir Marmor

**Affiliations:** 1 Department of Bioengineering and Therapeutic Sciences, University of California, San Francisco, CA 94158, USA; mike.morimoto@ucsf.edu (M.M.); rada.savic@ucsf.edu (R.S.); 2 School of Medicine, University of California, San Francisco, CA 94143, USA; ashraf.nawari@ucsf.edu; 3 Orthopaedic Trauma Institute, University of California, San Francisco, CA 94110, USA

**Keywords:** pain, wearables, machine learning

## Abstract

Poor pain alleviation remains a problem following orthopedic surgery, leading to prolonged recovery time, increased morbidity, and prolonged opioid use after hospitalization. Wearable device data, collected during postsurgical recovery, may help ameliorate poor pain alleviation because a patient’s physiological state during the recovery process may be inferred from sensor data. In this study, we collected smart ring data from 37 inpatients following orthopedic surgery and developed machine learning models to predict if a patient had postsurgical poor pain alleviation. Machine learning models based on the smart ring data were able to predict if a patient had poor pain alleviation during their hospital stay with an accuracy of 70.0%, an F1-score of 0.769, and an area under the receiver operating characteristics curve of 0.762 on an independent test dataset. These values were similar to performance metrics from existing models that rely on static, preoperative patient factors. Our results provide preliminary evidence that wearable device data may help control pain after orthopedic surgery by incorporating real-time, objective estimates of a patient’s pain during recovery.

## 1. Introduction

The significance of postoperative pain for patients following orthopedic surgery has been acknowledged as one of the primary factors affecting patient outcomes and quality of life [1]. In addition to decreased patient comfort, increased morbidity, and prolonged recovery [2], poor pain alleviation (PPA) after surgery increases the chances of chronic opioid use [3]. Despite the introduction of modern multi-modal pain alleviation regimens [4], PPA remains unacceptably high, with up to 46% of patients experiencing it in the two weeks following discharge from orthopedic surgery in the United States [1,4,5]. Therefore, reliably predicting patients’ pain levels postoperatively may be a meaningful way to detect and intervene in the development of complications from PPA, increase patient satisfaction, and enhance outcomes.

Many models of PPA prediction in the postoperative period have been created due to its importance in the development of potential complications. Almost all of these models rely on baseline factors that are non-modifiable during the recovery process, such as previous narcotic use, pain at baseline, body mass index (BMI), age, history of anxiety or depression, and tobacco use [6]. Though these existing PPA prediction models may help guide the need for enhanced postsurgical pain control, they lack the ability to react to changes in PPA that may occur during the recovery process. Inclusion of sensor data collected from the patient after surgery, which have shown to be correlated with pain in some cases [7], is a promising approach for objective and timely assessment of PPA and may help guide more personalized pain alleviation strategies. 

Non-invasive wearable devices are attractive candidates for postsurgical patient monitoring and collection of sensor data. Smart rings, in particular, have lightweight and compact form factors that may be comfortable for patients to wear and typically contain sensors from which a patient’s physiological status may be derived. For example, smart rings have been used in other studies to infer sleep quality [8], detect COVID-19 infection [9], and track menstrual cycles [10,11] from photoplethysmography (PPG), temperature, and acceleration sensors. These same sensors may be used to calculate physiological parameters that have been studied as correlates to pain [12,13,14], such as heart rate [15], heart rate variability [16,17], respiration rate [18], (lack of) activity [19], and deteriorated sleep quality [20,21,22]. Machine learning models can then be constructed to account for the potentially complex relationships between wearable sensor-derived metrics and pain-related endpoints [23,24].

While many studies have examined the relationship between wearable device data and pain-related endpoints [24,25], the use of wearable device data to predict PPA has not been studied in the acute postoperative setting of orthopedic surgery. This study, therefore, aims to use physiological smart ring device data, including cardiopulmonary rhythm, temperature, and activity data, to build prediction models that determine if a patient has adequate pain alleviation (APA) or PPA during the period following orthopedic surgery in a small patient population.

## 2. Materials and Methods

### 2.1. Study Design

This study was a prospective, non-interventional, observational study conducted at a single medical center. The enrollment target number was based on previous studies investigating the relationships between wearable device data and various physiological states, including sleep quality (sample size *n* = 45) [22], stress (*n* = 10 to *n* = 35) [13], and responses to opioid medications (*n* = 36) [25]. Since the stress response and pain may be correlated [14,26], and therefore may result in similar perturbations from normal wearable sensor data, an enrollment target of 45 participants was chosen to be similar to existing studies that use machine learning to estimate stress from wearable device data [13]. 

### 2.2. Participants

Inclusion criteria included English speaking adult (at least 18 years of age) inpatients undergoing hip and knee joint replacement surgery and were able to provide written consent. Prisoners, children, pregnant women, and patients with severe cognitive impairment that prevented them from following study directions were excluded; all other demographic information and patient characteristics were not used as exclusion criteria.

### 2.3. Wearable Device

Oura Ring smart rings were used for this study (Generation 3 Horizon model; Oura Health, Oulu, Finland). These devices had infrared PPG, temperature, and three-degrees-of-freedom acceleration sensors [27,28], from which metrics such as heart rate, body temperature, and activity may be estimated, respectively. The smart rings had battery capacities ranging from 15 mAh to 22 mAh (depending on ring size) and were able to store up to one week of data using onboard memory. The full list of the smart ring biometrics available for this study is provided in Table A1 in Appendix A.

### 2.4. Data Collection

Enrolled and consented patients were given a smart ring to wear postoperatively up until their time of their discharge. Patients were instructed to always keep the ring on, including during sleep. For each patient, data were automatically uploaded and synchronized to a research smartphone and then to the smart ring cloud server. These data were then subsequently downloaded as individual Excel files directly from the cloud for analysis. 

Electronic medical records (EMRs) were queried for data on surgery type, basic demographics, vital signs, pain medications administered, previous exposure to opioids, and nurse-recorded pain scores. Nurse-recorded pain scores were documented by verbally asking patients to rate their pain level on a 0-to-10 numeric rating scale (NRS). The information retrieved from EMRs was inputted into a REDCap database. Pain scores, pain medications, and vital signs were documented for each patient starting at 6:00 AM on the day after surgery until discharge. 

### 2.5. Data Processing and Modeling 

The majority of the smart ring data files provided daily summaries of metrics derived from sensor measurements for each day the device was worn, as opposed to minute-by-minute estimates of derived biometrics. Due to the more reliable availability of daily data, it was determined that patient days would represent the observations during subsequent model development. Since each patient stayed in the hospital for at least one day, using patient days also resulted in an increase in the total number of observations in the dataset.

The smart ring data files and EMR data comprised the model features explored in this analysis. All unique metrics from the smart ring daily data summaries were included, such as sleep-, readiness-, and activity-scores, and their respective “contributors”. For example, sleep score contributors included timing, deep sleep, restfulness, efficiency, latency, rapid eye movement sleep, and total sleep. The daily activity report included minute-by-minute activity estimates, from which summary statistical values were calculated and added as features. In addition to features derived from daily data summary reports, metrics from more granular data were extracted, such as summary statistical values for heart rate (HR) and heart rate variability (HRV) for each day. Finally, data from EMRs were added to the smart ring features, including basic patient demographics (age, weight, height, and gender), number of pain medications taken per day, and (if vital sign data were available) the average change in HR and HRV due to medications within a 2 h window per day. Each of the patient day observations were labeled according to whether PPA occurred on that day, with PPA defined as an NRS value ≥ 8 at least three times, separated by 4–12 h, during a 24 h period [29]. Univariate (mean value) imputation was used for missing data. Additional details on the data processing procedures are provided in Appendix A.

The relatively small number of observations required the investigation of feature selection methods and limited candidate classification models to be simple to help prevent overfitting. The feature selection methods investigated included recursive feature elimination (RFE) [30], Shapley additive explanations (SHAP) [31], and brute force combinations of feature subsets. As computational demand grows combinatorically in the number of features examined for the brute force feature selection methodology, model performance was used to provide a stopping criterion for the number of features chosen. The models evaluated were regularized logistic regression and XGBoost, with binary cross entropy used as the loss function during training. The models and training/evaluation pipelines were implemented using standard Python libraries [32,33,34] (see Appendix A for additional details). Alternative and more complex models were explored but suffered from overfitting due to the relatively small dataset used in this study.

The different feature selection and model structure combinations were evaluated on the training dataset using five-fold cross validation (CV), with average accuracy, F1-score, and area under the receiver operating characteristic curve (AUC ROC) across the folds used as metrics of model performance. Additionally, an inner five-fold CV was performed for each outer CV fold to optimize model hyperparameters, such as maximum iterations and the Elastic-Net mixing parameter for logistic regression models, or number of estimators and minimum loss reduction for XGBoost models. The best-performing feature selection and model structure combinations were then evaluated on an independent test dataset.

Model development and testing occurred simultaneously with patient enrollment. This allowed the modeling plan to incorporate strict out-of-sample testing, with the test dataset based on the last 10 enrolled patients and the training dataset derived from all earlier patients. Though this dataset split did not guarantee similarity between the training and testing datasets, and therefore risked poor performance during evaluation on the test dataset, it was decided that this approach was a more rigorous assessment of the modeling work due to the resulting guarantee of no leakage of the test dataset into the training process.

Though the models were developed on daily PPA predictions, per-patient PPA predictions were obtained by aggregating a patient’s daily PPA predictions into a single prediction. The mean value of per-day PPA predictions for each patient was chosen as the aggregation method for per-patient PPA predictions. The per-patient PPA predictions allowed for comparisons to be made with existing PPA models that are based on preoperative variables or demographic data. Though these existing models focused on finding statistically significant contributors to PPA rather than prediction of PPA, reported odds ratios can be used in logistic regression models for comparison to the developed smart ring-based models. These existing PPA models, developed using similar post-orthopedic surgery patient populations, were evaluated on the same testing dataset (last 10 enrolled patients) as the smart ring models. The training dataset was used to estimate non-reported model parameters, such as the intercept/baseline odds for logistic regression models (see Appendix A for additional details).

## 3. Results

A total of 102 patients were screened from 18 October 2022 to 14 November 2023, with 62 undergoing total knee replacement and 40 patients undergoing total hip replacement. Of these patients, 45 were enrolled, and all but 2 patients were not enrolled due to language barriers. The average length of stay for patients with completed data was 4.97 days, and the range of inpatient stay was 2–21 days. Of the 45 patients enrolled in the study, 37 successfully completed their smart ring data collection process. Demographics and characteristics of enrolled patients are provided in Table 1. 

The 37 patients that completed the data collection process had smart ring data from 110 days, with 75 features derived from the smart ring data files and five features from EMRs. The training dataset (patients 1–35) had 81 observations from 27 unique patients, whereas the testing dataset (patients 36–45) has 29 observations from 10 unique patients. The training and testing datasets had 27 (33%) and 12 (41%) daily observations that were associated with PPA, respectively. 

**Figure 1:**
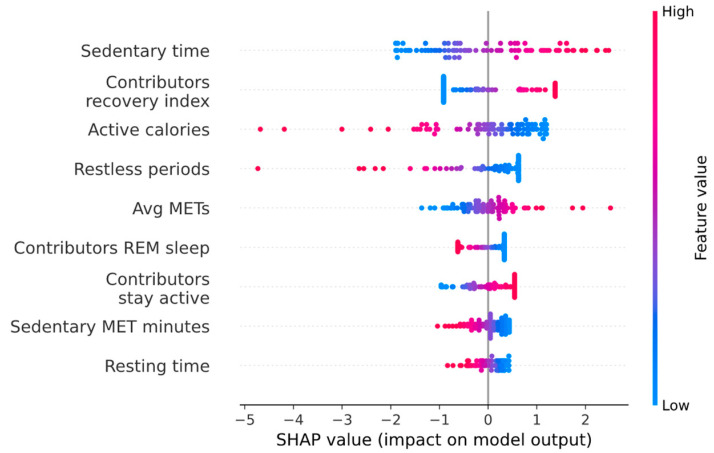
Features and behavior for the nine-feature brute force feature selection model on the training dataset (patients 1–35). The model behavior is represented as SHAP values and displays how each feature affects the probability of PPA.

Table 2 shows the feature selection and model structure combinations with the largest mean model performance metrics across the five training data CV folds. The brute force feature selection process was terminated after choosing nine features since the CV-averaged accuracy, F1-score, and AUC ROC appeared to plateau after choosing seven features. Though XGBoost models performed well on initial subsets of the training dataset (e.g., patients 1–25), overfitting led to generally poor performance on the CV validation datasets despite the inclusion and tuning of regularization parameters (L1, L2, and minimum loss reduction per split). Brute force feature selection produced feature subsets that generally achieved the highest CV-averaged accuracies and F1-scores, whereas the RFE and SHAP feature selection methodologies produced models with high CV-averaged ROC AUCs. An example of the features and model behavior of daily PPA predictions is shown in Figure 1 for the nine-feature brute force model. For this particular model, none of the EMR-derived features were selected, and all of the final features were procured from the smart ring data.

**Figure 2:**
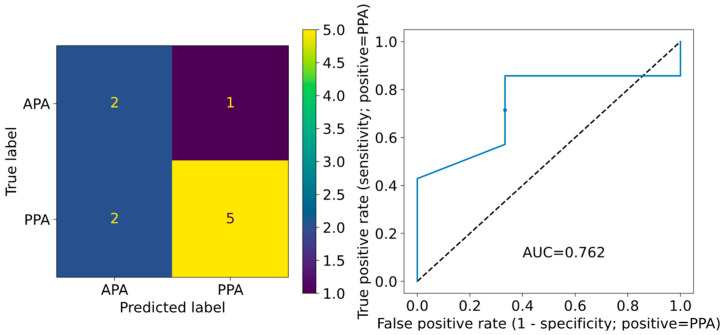
Test dataset (patients 36–45) results for aggregated per-patient PPA predictions using a logistic regression model with brute force feature selection with nine features. The model’s confusion matrix is on the left and ROC curve is on the right. The AUC ROC for the test dataset was 0.762, and the circular marker on the ROC curve depicts the location corresponding to a probability threshold of 0.5.

Table 2 also shows the model performance on the independent test dataset derived from the last 10 patients (patients 36–45) enrolled in the study. The results were generally poor, and even the best-performing model (brute force feature selection using nine features, illustrated in Figure 1) suffered from limited generalizability for prediction of PPA per day, with an accuracy of 58.6%, F1-score of 0.538, and AUC ROC of 0.612. Despite the poor daily PPA prediction performance, when the PPA per day predictions were mean-aggregated to PPA per-patient predictions, the best model performance improved to 70.0% accuracy, 0.769 F1-score, and 0.762 AUC ROC (see Figure 2).

**Figure 3:**
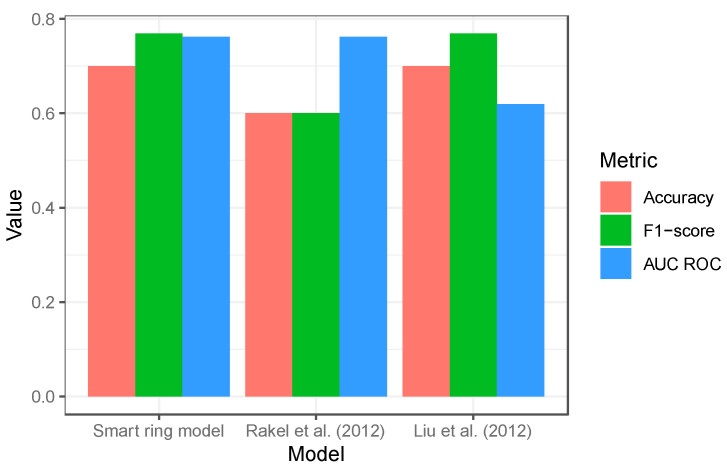
Comparison of smart ring model performance against existing models of post-orthopedic surgery PPA that rely on baseline/preoperative factors on the out-of-sample test dataset (patients 36–45). The models by Rakel et al. (2012) [35] and Liu et al. (2012) [36] relied on preoperative factors for predicting postoperative PPA. The discrepancies in AUC ROC for models that have similar accuracies and F1-scores (and vice versa) are due to differences in model-predicted probabilities of PPA.

In the context of existing models of PPA prediction, the aforementioned performance metrics can be compared to those obtained by evaluating models using published odds ratios for baseline/preoperative factors that were found to be predictors of postsurgical PPA. Two logistic regression models, developed using post-orthopedic surgery patient populations, were used for this comparison and were based on (1) preoperative pain, depression, and age for predicting postsurgical severe resting pain [35]; and (2) sex, age, BMI, surgery type (total hip or knee replacements), preoperative pain, preoperative opioid use, and general anesthesia for predicting moderate-to-severe postsurgical resting pain [36]. Figure 3 shows the performance of the smart ring model is similar to the two published models based on orthopedic surgery patients. 

## 4. Discussion

This study investigated the use of wearable device data in predicting acute PPA after orthopedic surgery. The results indicate that wearable sensor data can be used to predict PPA correctly for the majority of the patients in an out-of-sample, independent, test dataset. Considering that the wearable device data were collected at the same time as when PPA may have occurred, this study provides evidence that these data may reflect the physiological state of the wearer and opens the possibility of more individualized postsurgical pain control that is based on objective, real-time metrics.

The Oura Ring wearable device used in this study is a lightweight and non-invasive device worn on the finger and marketed to track activity, sleep, and heart rate. Though not designed to measure pain, quantities derived from its PPG, temperature, and acceleration sensors were shown to be related to PPA in the studied patient population. While other wearable devices have also been used for pain assessment, including wrist-worn actigraphy and HRV measurement devices [12], a smart ring has the advantages of patient comfort and ease of use due to its compact form factor; this was verified by the fact that none of the study participants indicated any issues or complaints that the wearable device interfered with their inpatient recovery process.

The best-performing models were logistic regression models with brute force feature selection. The higher performance of logistic regression models over alternative structures was largely due to the limited observations in the dataset, where the simpler model structure, despite not representing complex relationships between the features and PPA, helped to prevent overfitting. The higher performance of the brute force method over RFE and SHAP for feature selection was notable because it highlighted complex interactions between features when added or removed from a model. For example, the three features resulting in the best-performing logistic regression model were active calories, average metabolic equivalents (METs), and light sleep duration. When adding an additional feature, it may be expected that the best-performing set of four features would include the three aforementioned features; this was not, however, the case. This behavior, likely due to model misspecification, was not captured by the RFE or SHAP feature selection methods and provides an area for further investigation. 

The best-performing model, which was based on brute force feature selection and a logistic regression structure, is illustrated in Figure 1. All of the selected features were derived from the smart ring data, and none were from EMRs, providing evidence that the wearable sensor data during recovery may contain information related to a patient’s pain-related physiological state. Increased sedentary time, recovery index contribution, average METs, and stay active contribution increased the predicted probability of PPA; whereas increased active calories, restless periods, REM sleep contribution, sedentary MET minutes, and resting time decreased the predicted probability of PPA. Some of these dependencies are consistent with expected associations with PPA: a patient experiencing severe pain may spend more time being sedentary [37], expend fewer calories being active [38], and have an increased overall energy expenditure [39]. While other model dependencies may not have clear explanations and require additional investigation, it must also be considered that the feature values estimated by the smart ring are subject to error, resulting in difficult interpretations from a physiological perspective. 

The best performing daily PPA prediction model for all metrics on the test dataset was also the best-performing per-patient PPA prediction model for all metrics. This correspondence between daily and per-patient PPA predictions, though expected for well-performing models (i.e., perfect daily PPA predictions will also result in perfect per-patient PPA predictions), was not consistent for all models. Though daily PPA prediction F1-scores and AUC ROCs were both well-correlated to their respective per-patient PPA predictions (Pearson correlation coefficients of 0.901 and 0.955, respectively, both with *p*-values < 0.01), the accuracies for the two targets were not (Pearson correlation coefficient of 0.571 [*p*-value = 0.139]). These relationships between the daily and per-patient PPA prediction model metrics, which were likely affected by the small size of the test dataset in this study and the performance of the per-day PPA models, would be interesting to examine in a larger study population to optimize the prediction aggregation step.

The modeling results in this study provide preliminary evidence that smart ring data, collected during postsurgical recovery, is associated with PPA. Though the per-day PPA predictions were poor, the aggregated per-patient PPA predictive performance was similar to existing PPA models. The per-day aggregation to per-patient predictions, which allowed comparisons to existing models to be made, was further motivated by examining the features that were selected in the best-performing model (listed in Figure 1): it is possible that the effects of one day’s activity, sleep, and readiness spill over into subsequent days and consequently could be used to better understand a patient’s overall experience during recovery. Though not significantly better than existing PPA models based on baseline/preoperative factors, the smart ring model’s dependency on only postsurgical sensor data alludes to future possibilities of more accurate PPA predictions and real-time, objective estimates of a patient’s pain after integrating next-generation models into a wearable device. An exciting consequence is that physiological responses to pain medications can be measured and used for more effective pain alleviation therapies. Similar to the use of data monitors to improve control of anesthesia depth [40], the precision medicine offered by sensor-aided control of postoperative pain may significantly improve the patient’s experience following orthopedic surgery.

This study is the first to focus on using smart ring device data for personalized pain estimation within the orthopedic surgery population. It demonstrates the potential for real-time and objective measures of pain during the postoperative period and helps to motivate further research on this topic. However, this study has some noteworthy limitations. Most importantly, despite enrolling for over a year and screening 102 patients, only 45 patients were enrolled, and only 37 patients successfully completed the smart ring data collection process. This small sample size, combined with the fact that enrollees were from a single medical center, may limit the generalizability of the results to a broader population. Additionally, because the smart ring device was not specifically designed for assessing pain, it was only possible to model correlations between the available smart ring metrics and postoperative PPA. The reliance on only the available smart ring metrics may have prevented an examination of raw sensor signals and patterns that were better associated with PPA, which may have resulted in better predictive performance of the models developed in this study. Finally, the decision to have true out-of-sample testing using the last-enrolled patients, rather than split the training and testing datasets using stratification, may have decreased the reported performance metrics due to dissimilarities between the training and testing datasets. Table 1 shows that the testing dataset has many patient characteristics that are dissimilar from those in the training dataset.

## 5. Conclusions

This study provides preliminary evidence that PPA may be predicted using wearable device data following orthopedic surgery, which has the advantage of reflecting a patient’s physiological status during recovery. In contrast to static baseline patient data that are currently used to predict PPA, wearable device data may allow for more accurate real-time interventions for addressing patients’ pain levels. Given the increasing focus on multimodal pain regimens in orthopedics, wearable device data reflecting physiological status could potentially narrow the scope of pain regimens and decrease the burden of polypharmacy on patients. Having said this, further investigation is needed to improve model performance through larger patient samples, along with exploration of different wearable device biometrics followed by clinical validation studies to determine the extent to which wearable device data can be consistently used in clinical settings for pain assessment in the acute postoperative period.

## Figures and Tables

**Table 1:** Patient demographics and characteristics for all patients and the datasets used in the smart ring PPA models.

	All Patients	Train Dataset	Test Dataset
Patients, *n*	45	27	10
PPA per patient, *n* (%)	20 (44)	13 (48)	7 (70)
Sex, female, *n* (%)	24 (53)	12 (44)	5 (50)
Age, year			
Median	59	59	58
IQR	54–65	54–64	55–66
Range	28–82	28–78	51–82
Body weight, kg			
Median	86	80	100
IQR	68–110	66–110	76–120
Range	38–179	38–175	58–179
BMI, kg/m^2^			
Median	30.6	28.7	36.5
IQR	25.5–36.7	24.9–35.5	30.0–37.7
Range	11.4–75.7	11.4–75.7	23.9–49.6
ASA physical status score			
Median	3	3	3
IQR	2.0–3.0	2.0–3.0	3.0–3.0
Range	1.0–3.0	1.0–3.0	2.0–3.0
Comorbidities, *n* (%)	32 (71)	22 (81)	5 (50)
Depression or anxiety, *n* (%)	15 (33)	7 (26)	4 (40)
Smoker, *n* (%)	10 (22)	5 (19)	3 (30)
Previous opioid use, *n* (%)	36 (80)	22 (81)	10 (100)
Baseline pain score			
Median	5.5	7	0
IQR	0.0–8.0	0.0–8.0	0.0–6.5
Range	0.0–10.0	0.0–10.0	0.0–10.0

Abbreviations: IQR: interquartile range; ASA: American Society of Anesthesiologists.

**Table 2:** Training and testing results for models developed using smart ring data.

Model Details			Model Performance								
			Pt 1–35 Training Dataset *			Pt 36–45 Testing Dataset (Daily PPA)			Pt 36–45 Testing Dataset (Aggregated Per-Patient PPA)		
Structure	Feature Selection Method	Number of Features	Accuracy	F1-Score	AUC ROC	Accuracy	F1-Score	AUC ROC	Accuracy	F1-Score	AUC ROC
Logistic regression	RFE	28	0.715	0.567	0.761	0.552	0.316	0.534	0.400	0.250	0.667
Logistic regression	SHAP	18	0.720	0.623	0.840	0.552	0.316	0.486	0.500	0.444	0.429
Logistic regression	Brute force	4	0.757	0.483	0.740	0.483	0.118	0.203	0.300	0.222	0.143
Logistic regression	Brute force	5	0.785	0.642	0.740	0.517	0.125	0.203	0.400	0.250	0.143
Logistic regression	Brute force	6	0.785	0.642	0.745	0.517	0.125	0.203	0.400	0.250	0.143
Logistic regression	Brute force	7	0.795	0.622	0.783	0.517	0.461	0.534	0.700	0.769	0.571
Logistic regression	Brute force	8	0.788	0.631	0.780	0.517	0.300	0.593	0.500	0.545	0.524
Logistic regression	Brute force	9	0.802	0.625	0.757	0.586	0.538	0.612	0.700	0.769	0.762

* Average values across five CV folds. Abbreviations: AUC ROC: area under the receiver operating characteristics curve.

**Figure A1:**
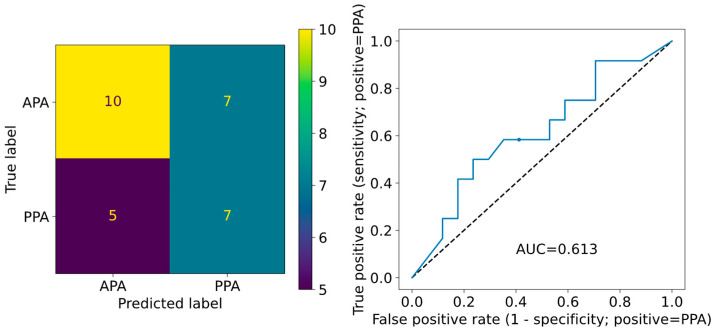
Test dataset (patients 36–45) results for per-day PPA predictions using a logistic regression model with brute force feature selection with nine features. The model’s confusion matrix is on the left and ROC curve is on the right. The AUC ROC for the test dataset was 0.613, and the circular marker on the ROC curve depicts the location corresponding to a probability threshold of 0.5.

## Data Availability

The dataset is available on request from the authors.

## Appendix A

### Appendix A.1. Smart Ring Features

The smart ring features were contained in two types of data files: daily reports and more detailed heart rate and sleep reports. The daily reports provided summary metrics for each day the smart ring was worn and provided most of the features used. The more detailed heart rate report listed time-stamped heart rate values for periods of time when the smart ring determined reliable heart rate values could be estimated. The detailed sleep report provided various metrics for every period when the smart ring determined the patient was sleeping, including heart rate (HR) and heart rate variability (HRV). These were used to calculate the “delta HR” and “delta HRVRR” features, which represented the average change in vital sign metric within 2 h windows before and after pain medication administration. Note that these “delta” features are not always available for medication doses because the HR and HRV quantities were not regularly calculated/sampled.

All features used in the smart model development are listed in Table A1. In the Table, a “contributor” is a factor that is used to calculate a sleep/activity/readiness score. “HRVRR” is HRV divided by (daily averaged) respiration rate, a quantity that was initially expected to be correlated with acute pain. Repeated features (e.g., reported in both the daily and more detailed sleep reports) were removed.

**Table A1:** Training and testing results for models developed using the smart ring data.

Feature	Source	Feature	Source
Active calories	Oura Ring	Meters to target	Oura Ring
Average breath	Oura Ring	Movement 30 s	Oura Ring
Average heart rate	Oura Ring	Non wear time	Oura Ring
Average HRV	Oura Ring	Period	Oura Ring
Average MET minutes	Oura Ring	Readiness contributors body temperature	Oura Ring
Awake time	Oura Ring	Readiness contributors previous day activity	Oura Ring
Contributors activity balance	Oura Ring	Readiness contributors previous night	Oura Ring
Contributors body temperature	Oura Ring	Readiness contributors recovery index	Oura Ring
Contributors deep sleep	Oura Ring	Readiness score	Oura Ring
Contributors efficiency	Oura Ring	Readiness temperature deviation	Oura Ring
Contributors HRV balance	Oura Ring	Readiness temperature trend deviation	Oura Ring
Contributors latency	Oura Ring	Rem sleep duration	Oura Ring
Contributors meet daily targets	Oura Ring	Resting time	Oura Ring
Contributors move every hour	Oura Ring	Restless periods	Oura Ring
Contributors previous day activity	Oura Ring	Sedentary MET minutes	Oura Ring
Contributors previous night	Oura Ring	Sedentary time	Oura Ring
Contributors recovery index	Oura Ring	Sleep midpoint	Oura Ring
Contributors recovery time	Oura Ring	Steps	Oura Ring
Contributors REM sleep	Oura Ring	Target calories	Oura Ring
Contributors restfulness	Oura Ring	Target meters	Oura Ring
Contributors resting heart rate	Oura Ring	Temperature deviation	Oura Ring
Contributors sleep balance	Oura Ring	Temperature trend deviation	Oura Ring
Contributors stay active	Oura Ring	Time in bed	Oura Ring
Contributors timing	Oura Ring	Total calories	Oura Ring
Contributors total sleep	Oura Ring	Total sleep duration	Oura Ring
Contributors training frequency	Oura Ring	Avg METs	Oura Ring
Contributors training volume	Oura Ring	Max METs	Oura Ring
Deep sleep duration	Oura Ring	Activity score	Oura Ring
Efficiency	Oura Ring	Readiness score	Oura Ring
Equivalent walking distance	Oura Ring	Daily sleep score	Oura Ring
High activity MET minutes	Oura Ring	Sleep score	Oura Ring
High activity time	Oura Ring	N med doses	EMR
Inactivity alerts	Oura Ring	Delta HR	Oura Ring
Latency	Oura Ring	Delta HRVRR	Oura Ring
Light sleep duration	Oura Ring	Age	EMR
Low activity MET minutes	Oura Ring	Weight	EMR
Low activity time	Oura Ring	Height	EMR
Lowest heart rate	Oura Ring	Gender female	EMR
Medium activity MET minutes	Oura Ring	Gender male	EMR
Medium activity time	Oura Ring	Gender other	EMR

### Appendix A.2. Feature Processing, Imputation, and Selection

Many features had missing values. As an example, there were many instances where the smart ring was not able to collect sufficient data to allow calculation of the average change in HR around a medication administration time, resulting in several of the “delta HR” feature values missing. For the combined training and testing datasets, there were 2331 (26.5%) missing features. These missing values were imputed since many model types cannot handle missing values natively (i.e., logistic regression).

Highly correlated features (Pearson correlation coefficient > 0.9) were removed prior to feature imputation. Since the presence of missing data may provide useful information (e.g., the smart ring determining that HR could not be calculated because of high activity levels), the value −1 was substituted for features that had positive values. For the remaining missing features, several imputation strategies were explored in an interim analysis, including multivariate Bayesian regression and nearest neighbor approaches, but the univariate mean imputation strategy was carried forth because it generally produced the best averaged cross validation (CV) performance metrics on the interim analysis dataset.

Feature selection was performed by recursive feature elimination (RFE), Shapley additive explanations (SHAP), and brute force feature combinations. For the RFE and SHAP attribution methods, features were rank-ordered according to their importance, and feature groups were constructed from the top ranked features. These feature groups, which, for a given number of features *m*, was a subset of all feature groups with *m* + *k* features (*k* > 0), were then evaluated to find the optimal number of features to use. This was based on the average accuracy across the CV folds, with the optimal feature group having the highest average accuracy. The brute force feature selection method differed from the RFE and SHAP methods because the candidate features were not rank-ordered, and smaller feature groups were not necessarily subsets of larger feature groups.

### Appendix A.3. Modeling

The modeling pipeline consisted of standardization of feature values, imputation of missing feature values, standardization of feature values again, and either a regularized (Elastic Net) logistic regression or XGBoost model. “Outer” CV folds were used to find the best feature and model structure combinations, and “inner” CV folds (derived from the outer CV training data) were used to tune model hyperparameters. A grid search was used to find the optimal combination of the following hyperparameters for each model:

- Logistic regression: L1:L2 ratio, whether or not to apply class weights, whether or not to fit an intercept, and the maximum number of iterations.
- XGBoost: number of estimators, maximum depth, lambda, alpha, eta, and gamma.

Analyses were performed using Python 3.9.

### Appendix A.4. Results

The confusion matrix and receiver operating characteristics (ROC) curve for the best-performing model (logistic regression with brute force feature selection using nine features) are provided in Figure A1, which shows the results for the per-day PPA predictions. The aggregated per-patient PPA prediction confusion matrix and ROC curve is provided in the main text.

### Appendix A.5. Comparison to Other Models

Existing models of poor control of pain following orthopedic surgery typically focus on finding significant predictors of pain, where statistical significance means that the model parameter associated with a feature has a value different from zero. In this framework, odd ratios are commonly reported, from which logistic regression model parameters may be obtained by applying a natural logarithm.

A model developed by Rakel et al. (2012) [35] examined baseline/preoperative factors that were associated with severe postsurgical resting pain after total knee replacement procedures using data from 179 patients. A logistic regression model was developed and odds ratios for preoperative resting pain, depression, and age were reported. It was assumed that an intercept was fitted to the data (baseline odds), which was estimated using data from patients 1–35 in the current study’s patient population.

A model developed by Liu et al. (2012) [36] examined baseline/preoperative factors that were associated with moderate-to severe postsurgical resting pain after total knee or hip replacement procedures using data from 897 patients. A generalized estimating equation model was developed and odds ratios for gender, age, body mass index, surgery type (knee or hip replacement), preoperative pain at surgical site, preoperative use of opioids, and anesthesia type (general or local) were reported. No information on the correlation structure for the generalized estimating equations was reported, though it could be inferred that observations were grouped according to study site. Since the current study recruited patients from a single study site, a simple linear regression model structure was assumed to represent the relationship between the aforementioned variables and postsurgical pain. It was assumed that an intercept was fitted to the data (baseline odds), which was estimated using data from patients 1–35 in the current study’s patient population.
